# Supplementary material for: Novel Driver Strength Index highlights important cancer genes in TCGA PanCanAtlas patients
Source: PeerJ. 2022 Aug 11;10:e13860. doi: 10.7717/peerj.13860 (PMC9375969; doi:10.7717/peerj.13860)
Supplement: Supplemental Information 2 [file peerj-10-13860-s002.zip › COHORTS/genes plots/detailed/2021_8_16_14_9_distribution_events_detailed_6.pdf]

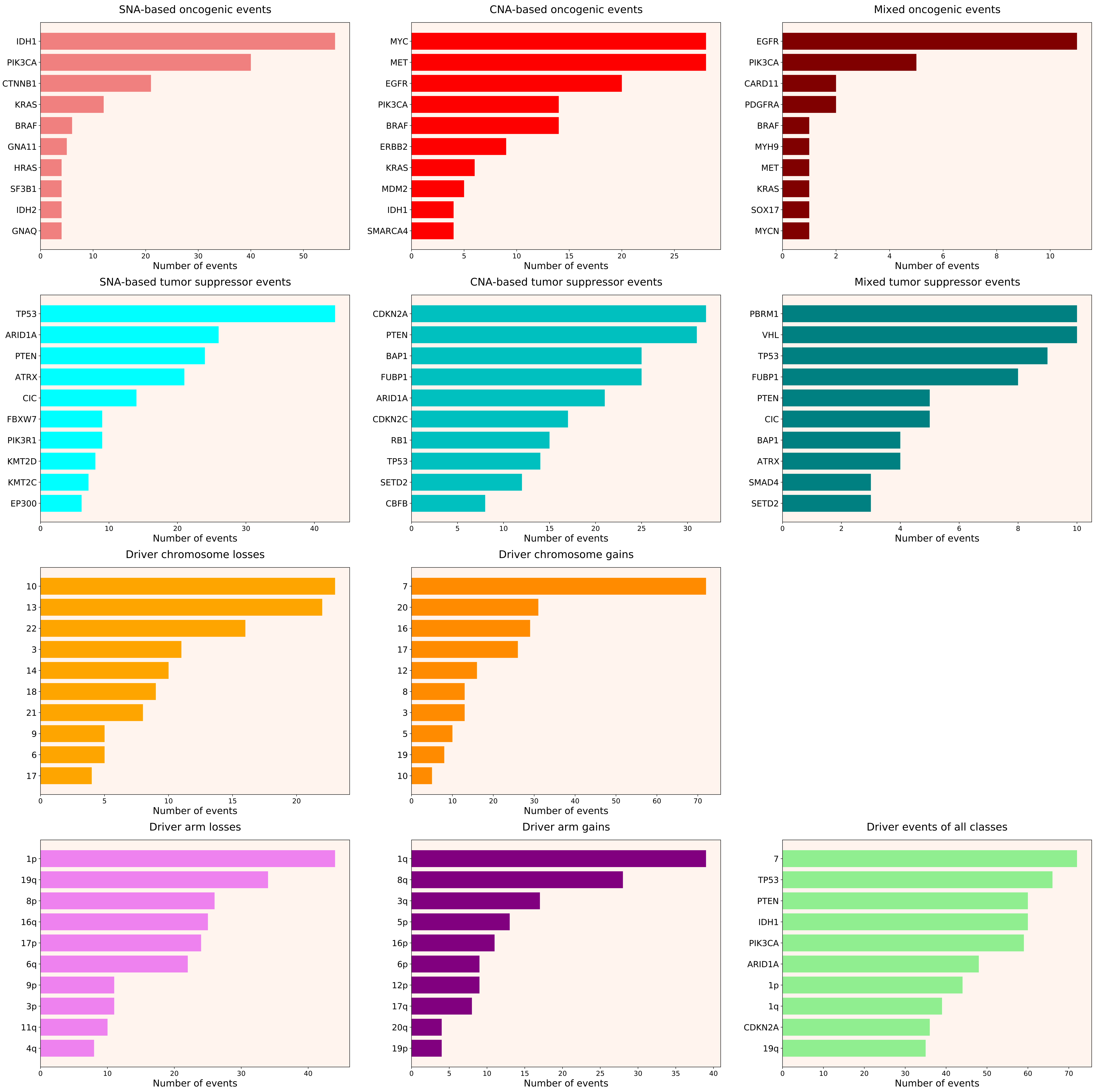
SNA-based oncogenic events
IDH1
PIK3CA
CTNNB1
KRAS
BRAF
GNA11
HRAS
SF3B1
IDH2
GNAQ
0 10 20 30 40 50
Number of events
CNA-based oncogenic events
MYC
MET
EGFR
PIK3CA
BRAF
ERBB2
KRAS
MDM2
IDH1
SMARCA4
0 5 10 15 20 25
Number of events
Mixed oncogenic events
EGFR
PIK3CA
CARD11
PDGFRA
BRAF
MYH9
MET
KRAS
SOX17
MYCN
0 2 4 6 8 10
Number of events
SNA-based tumor suppressor events
TP53
ARID1A
PTEN
ATRX
CIC
FBXW7
PIK3R1
KMT2D
KMT2C
EP300
0 10 20 30 40
Number of events
CNA-based tumor suppressor events
CDKN2A
PTEN
BAP1
FUBP1
ARID1A
CDKN2C
RB1
TP53
SETD2
CBFB
0 5 10 15 20 25 30
Number of events
Mixed tumor suppressor events
PBRM1
VHL
TP53
FUBP1
PTEN
CIC
BAP1
ATRX
SMAD4
SETD2
0 2 4 6 8 10
Number of events
Driver chromosome losses
10
13
22
3
14
18
21
9
6
17
0 5 10 15 20
Number of events
Driver chromosome gains
7
20
16
17
12
8
3
5
19
10
0 10 20 30 40 50 60 70
Number of events
Driver arm losses
1p
19q
8p
16q
17p
6q
9p
3p
11q
4q
0 10 20 30 40
Number of events
Driver arm gains
1q
8q
3q
5p
16p
6p
12p
17q
20q
19p
0 5 10 15 20 25 30 35 40
Number of events
Driver events of all classes
7
TP53
PTEN
IDH1
PIK3CA
ARID1A
1p
1q
CDKN2A
19q
0 10 20 30 40 50 60 70
Number of events
